# Overcoming Steroid Resistance in T Cell Acute Lymphoblastic Leukemia

**DOI:** 10.1371/journal.pmed.1002208

**Published:** 2016-12-20

**Authors:** Steven Goossens, Pieter Van Vlierberghe

**Affiliations:** 1 Center for Medical Genetics, Ghent University Hospital, Ghent, Belgium; 2 Department for Biomedical Molecular Biology, Ghent University, Ghent, Belgium; 3 Cancer Research Institute Ghent (CRIG), Ghent, Belgium

## Abstract

In a Perspective, Pieter Van Vlierberghe and Steven Goossens discuss Meijerink and colleagues' findings on steroid resistance in pediatric T cell acute lymphoblastic leukemia.

T cell acute lymphoblastic leukemia (T-ALL) is an aggressive hematological cancer that is mainly diagnosed in children and arises from the malignant transformation of T cell progenitors [1]. Current treatment protocols consist of high-dose, multi-agent combination chemotherapy and result in cure rates of greater than 85% in children. However, despite their clinical success, the toxic nature of these aggressive treatment regimens causes severe side effects such as reduced intellectual capacity, osteonecrosis and growth deficiencies, infertility, and an increased risk of secondary tumor development later in life. Therefore, integration of novel targeted therapies into contemporary T-ALL treatment protocols will be required to increase the quality of life for pediatric T-ALL survivors [2].

Glucocorticoids, such as prednisone or dexamethasone, are core components of the high-dose chemotherapy schedules used for the treatment of T-ALL [3]. The apoptotic response induced by these glucocorticoids is mediated by their interaction with the glucocorticoid receptor NR3C1 (Fig 1). Upon ligand binding, NR3C1 undergoes dimerization and translocates to the nucleus in order to regulate target gene expression through direct interaction with a specific DNA binding motif (glucocorticoid response element) [3]. One important glucocorticoid target gene is the pro-apoptotic BCL2 family member *BCL2L11* (encoding the BIM protein), which counteracts the anti-apoptotic activity of BCL2, BCL-XL, and MCL1 and will trigger a steroid-induced apoptotic response in steroid-sensitive leukemic blasts (Fig 1). Nevertheless, glucocorticoid response is variable amongst T cell leukemia patients, and steroid resistance has been associated with an increased risk of relapse and poor clinical outcome [3]. Therefore, increased understanding of the molecular mechanisms that drive glucocorticoid resistance in human T-ALL could trigger the development of novel strategies that restore steroid sensitivity in this disease. Ultimately, these efforts should improve clinical outcome in human T-ALL and reduce the detrimental toxic side effects associated with high-dose chemotherapy.

**Fig 1:**
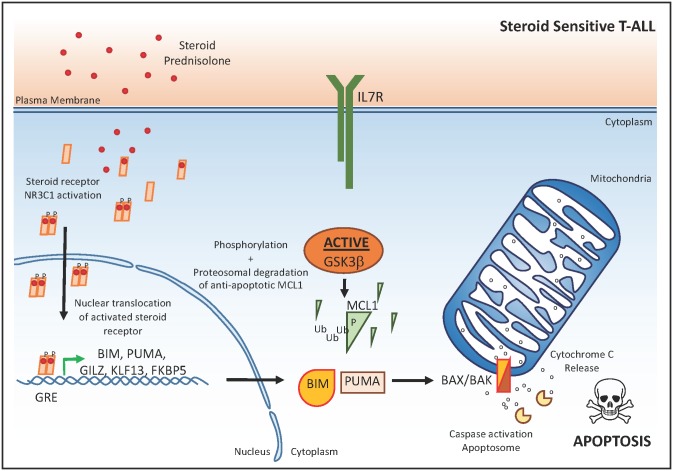
The glucocorticoid response in T-ALL. In steroid-sensitive T-ALL cells, steroid-mediated activation, dimerization, and subsequent nuclear translocation of the steroid receptor NR3C1 results in transactivation of the pro-apoptotic BIM and PUMA, which will activate the apoptosome complex after cytochrome C release from the mitochondria. The anti-apoptotic protein MCL1 in steroid-sensitive T-ALL cells is actively targeted for proteasomal degradation via GSK3β. βP, phosphorylation; Ub, ubiquination; GRE, glucocorticoid response element.

## Genetics Meet Drug Sensitivity in Human T-ALL

In this issue of *PLOS Medicine*, Jules Meijerink and colleagues [4] integrated next-generation sequencing data and genome-wide copy number profiles with ex vivo drug sensitivity and clinical outcome in a large panel of primary T-ALL specimens obtained at diagnosis. Interestingly, the authors found that mutations in the interleukin 7 receptor (IL7R) signaling pathway, including *JAK1* and *KRAS* mutations, were significantly associated with prednisolone resistance and reduced survival in an initial discovery cohort of 69 primary T-ALL samples. Of note, these results are in line with the poor prognostic implications of *JAK1* [5] and *N/K-RAS* [6] mutations that have previously been reported in adult T-ALL. In addition, previous studies have also shown an association between *RAS* mutations and steroid resistance in the context of precursor B-lineage ALL (B cell acute lymphoblastic leukemia [B-ALL]) [7,8]. Given that various mutations affecting the IL7R signaling cascade have also been reported in precursor B-ALL, it is tempting to speculate that the relation between IL7R signaling and steroid resistance could be a general feature of both T- and B-lineage ALL.

Next, Meijerink and colleagues extended their analyses to 146 leukemia samples, for which they determined the mutational status of a selected panel of genes required for IL7R–RAS–MAPK–AKT signaling (*IL7Ra*, *JAK1*, *JAK3*, *NF1*, *NRAS*, *KRAS*, and *AKT*) by PCR-based Sanger sequencing. This analysis revealed that T-ALL specimens mutated for any of these genes were significantly associated with steroid resistance and poor clinical outcome. Interestingly, T-ALLs that harbor mutations in IL7R–RAS–MAPK–AKT signaling were associated with specific molecular genetic subtypes, including immature, *TLX1*, *TLX3*-rearranged, or *HOXA*-activated leukemias. However, it should be noted that Meijerink and colleagues are the first to establish that these specific genetic alterations are truly associated with steroid resistance in primary T-ALL specimens.

## From Association to Functional Implications: Genetic Drivers of Steroid Resistance

**Fig 2:**
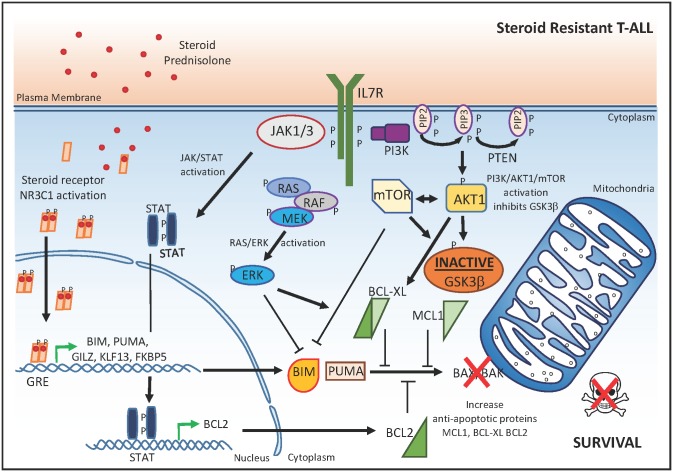
Molecular mechanisms that drive glucocorticoid resistance in T-ALL. In steroid-resistant T-ALL cells, the JAK/STAT, RAS/pERK, and PI3K/AKT1/mTOR signaling pathways result in: (1) up-regulation of the anti-apoptotic proteins BCL2 and BCL-XL; (2) inactivation of GSK3β, leading to increased concentrations of the anti-apoptotic protein MCL1; and (3) direct inhibition of BIM, which collectively counteract the NR3C1-mediated activation of the apoptosome complex. P, phosphorylation; GRE, glucocorticoid response element.

To functionally validate the putative contribution of each of these mutations to steroid resistance in T-ALL, Meijerink and colleagues generated a panel of isogenic tumor lines with inducible expression of IL7R–RAS–MAPK–AKT pathway mutations. Using these elegant in vitro model systems, the authors confirmed that *IL7R*, *JAK1*, and *NRAS* mutations, as well as overexpression of wild-type NRAS or AKT, could drive steroid resistance in human T-ALL cell lines (Fig 2). In contrast, no effect was observed for mutant JAK3 or expression of wild-type JAK1, JAK3, or IL7R.

Notably, no differences in NR3C1 nuclear translocation or target gene transactivation upon glucocorticoid exposure were observed between steroid-resistant and steroid-sensitive isogenic tumor cell lines. These data suggest that, at least in these in vitro model systems, the molecular mechanisms controlling steroid resistance are downstream or independent of the NR3C1 transactivation response. Strikingly, these results are in sharp contrast with other work in which glucocorticoid resistance in ALL was associated with the inability to induce the pro-apoptotic NR3C1 target gene *BCL2L11* upon dexamethasone treatment of patient-derived xenografts [9,10]. These contradictory results may be related to a differential epigenetic state of the *BCL2L11* (BIM) locus in the T-ALL cell lines versus patient-derived xenografts. In addition, these results suggest that steroid resistance can be gained via various means at different essential nodes in these converging signaling pathways.

Instead of a dysfunctional glucocorticoid-induced transcriptional response, Meijerink and colleagues identified aberrant activation of specific signaling pathways downstream of mutant IL7R, JAK1 or NRAS, or wild-type AKT as major determinants of steroid resistance in the isogenic tumor lines (Fig 2). Indeed, expression of *IL7R*, *JAK1*, or *NRAS* mutations provoked a strong activation of MEK–ERK and AKT signaling with concomitant phosphorylation of p70-S6K and an increased expression of anti-apoptotic BCL-XL. In addition, activated MEK–ERK and AKT signaling also triggered a robust inactivation of GSK3β, which would lead to stabilization of anti-apoptotic MCL1 (Fig 2). Interestingly, the association between IL7R–JAK signaling and steroid resistance described in this study is in line with the interleukin 7-induced anti-apoptotic rescue of primary T-ALLs against dexamethasone, as reported more than a decade ago [11]. However, in these studies, the inhibition of apoptosis by interleukin 7 was shown to correlate with up-regulation of BCL2 [12]. In addition, recurrent mutations in the IL7R and RAS signaling pathways mainly occur in early immature T-ALLs, which are also more often characterized by increased concentrations of BCL2 [13,14].

## Small Molecules with a Future in Steroid-Resistant T-ALL

**Fig 3:**
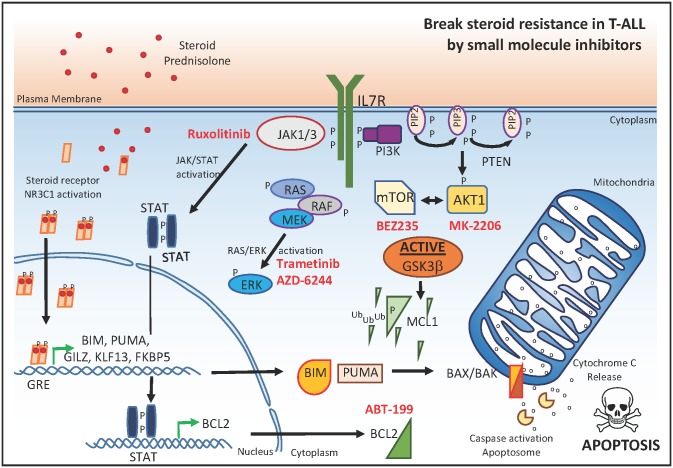
Small molecule inhibitors that reverse glucocorticoid resistance in T-ALL. Small molecules targeting JAK1, MEK1/2, or PI3K/AKT/mTOR (marked in red) in synergy with glucocorticoids can serve as a useful strategy to overcome steroid resistance in the context of malignant T cell transformation. P, phosphorylation; Ub, ubiquination; GRE, glucocorticoid response element.

Meijerink and colleagues’ study has unraveled specific pathways that influence glucocorticoid sensitivity in T-ALL by affecting the balance between pro- and anti-apoptotic factors. Therefore, specific targeting of these signaling cascades could provide a unique opportunity to overcome glucocorticoid resistance in human T-ALL. Indeed, the JAK1 inhibitor ruxolitinib, the MEK inhibitor CI1040, and the AKT inhibitor MK2206 were able to reverse steroid resistance in specific isogenic tumor lines (Fig 3). Interestingly, the authors also showed that the GSK3β inhibitor IX provoked strong steroid resistance in sensitive T-ALL tumor lines, further confirming the pivotal role of this molecule in the regulation of glucocorticoid resistance. Moreover, given the central role for the balance between pro- and anti-apoptotic factors in this process, one could hypothesize that specific targeting of the apoptotic machinery using BCL2 [13] or MCL1 [15] inhibitors could also serve as a useful strategy to alter steroid resistance in the context of malignant T cell transformation.

In a more clinically relevant approach, the authors subsequently evaluated the putative drug synergism between prednisolone and seven small molecule inhibitors targeting JAK1, MEK1/2, or PI3K/AKT/mTOR in a panel of 11 genetically well-characterized primary T-ALL samples at diagnosis. Notably, small molecule inhibitors targeting MEK–ERK or PI3K/AKT/mTOR signaling enhanced the steroid response in most primary T-ALL samples (Fig 3). In line with this notion, recent work showed that the dual PI3K/AKT/mTOR inhibitor BEZ235 enhanced dexamethasone sensitivity in vivo using a patient-derived T-ALL xenograft [16]. Surprisingly, and in contrast with the cell line data, ruxolitinib had a synergistic effect on prednisolone treatment in only 1 out of 11 primary T-ALL samples. However, this might be related to the lack of proliferative capacity of T-ALL cells in this system, given that previous work convincingly showed preclinical in vivo efficacy of ruxolitinib in patient-derived T-ALL xenografts [14]. Nevertheless, these results could suggest that ruxolitinib might not be suitable for therapeutic targeting of dormant leukemic stem cells.

In conclusion, Meijerink and colleagues’ study nicely illustrates how somatic alterations implicated in T-ALL disease biology influence the differential steroid response observed in the clinic. These novel insights strongly suggest that inhibition of MEK–ERK or PI3K/AKT/mTOR signaling could enhance steroid sensitivity in T-ALL and potentially improve patient treatment outcome, a notion that warrants further investigation in future prospective clinical trials.

## Abbreviations

B-ALL: B cell acute lymphoblastic leukemia
IL7R: interleukin 7 receptor
T-ALL: T cell acute lymphoblastic leukemia
